# An Account of a Remakable Disease of the Heart

**Published:** 1786

**Authors:** Nicholas Chavasse

**Affiliations:** Surgeon at Walfall, in Staffordshire, and Member of the Corporation of Surgeons of London


V. *An Account of a remakable Disease of the Heart. Communicated in a Letter to Dr.* Simmons *by Mr.* Nicholas Chavasse, *Surgeon at Walsall, in Staffordshire, and Member of the Corporation of Surgeons of London.*

THE Rev. Noah Jones, aged sixty-one years, had for a succession of years suffered extreme distress from a dyspnœa and violent cough, which he was of opinion had originated from repeated attacks of fever. He frequently expectorated large quantities of purulent matter, and observed, that, in an atmosphere moderately warm, he constantly spit more freely than in winter, when the weather was intensely cold; at which season his respiration was less laborious. For several years he had been emaciated to an extreme degree; and from the appearance of the matter expectorated, from the difficulty of breathing, and loss of muscular flesh, his case had been treated as a pulmonary consumption; and indeed the sole objection to the supposition was, the remarkable slowness of his pulse, and the absence of colliquative evacuations from the commencement to the conclusion of the disease. A palpitation of the heart upon accelerated motion was the only circumſtance that ſeemed to indicate effuſion in the cavity of the thorax. He ſlept as well on the right as on the left ſide; and, upon relating his caſe to me, he aſſured me, that he had never ſuffered much pain in his cheſt. A few months before his death he became hoarſe, and had nearly loſt his voice before that event took place. In a converſation I held with him a ſhort time before his diſſolution, he expreſſed a poſitive conviction that he ſhould die ſuddenly. He ſpoke peremptorily and chearfully on the ſubject; indeed he had no cauſe to be intimidated at ſuch a change, for his life had invariably been a pattern of ſtrict integrity, rigid temperance, and benevolent charity. As his diſeaſe was ſingular in its progreſs, and as its event was ſuch as he himſelf had conceived, I was deſirous of examining the thoracic viſcera, to aſcertain what morbid changes a diſeaſe of near twenty years duration had occaſioned in organs ſo neceſſary to animal exiſtence as the lungs and heart. The following may be relied on as a faithful relation of the ſtate of the parts I found in the cheſt: — I was not allowed ſufficient time to be particular in examining the abdominal viſcera.

Upon diſſecting the integuments from the ſternum num and ribs, in the usual manner, I was surprised to find scarce a vestige of the intercostal muscles; for these, as well as the pectoral muscles, &c. had much more the appearance of tendinous substance than of red flesh. The cartilages that connect the ribs to the sternum were more or less ossified; indeed the two inferior ones, on the left side, were evidently become bone, there not being the most distant resemblance in them to cartilaginous matter. On raising the sternum, the lungs were found partially adherent to the pleura on both sides; and, upon making incisions in different parts of the lobes, I found great solution of continuity, occasioned by ulcerated tubercles. This ulceration had considerably affected different parts of the trachea, especially on its superior part, the glottis being entirely occupied by an ill-conditioned sore, and the epiglottis appearing to be in a state of violent inflammation. The pericardium appeared less than usual, and its inferior and posterior part was connected to the heart itself by a membranous chord. About five ounces and an half of bloody serum, which coagulated on being exposed to the action of the fire, were effused in its cavity: but the most remarkable change seemed to have taken place in

 the the heart itſelf. Its ſize was ſmaller than that of an infant newly born. Its inveſting membrane was covered with a depoſit of bony matter, on the inferior part of the left ventricle, of nearly the extent of a ſhilling, and on the edge of this oſſification we diſcovered a rupture ſufficiently large to admit the introduction of a common probe. The muſcular fibres of this viſcus, like thoſe of the other parts, had more the appearance of ligamentous matter than of red fleſh, and were ſeparated upon the leaſt force being employed. Coagulated polypi were obſerved as uſual; but theſe I preſume were rather the conſequence of death than the cauſe of the diſeaſe. I had occaſion to remark, that the ulceration was confined to the veſicular part of the lungs, for, in different places, ſeveral ramifications of the pulmonary vein and artery were left expoſed, from having been deprived of their ſpongy covering. I diſcovered nothing remarkable in the abdomen, but a cartilaginous appearance on the peritoneal covering of the ſuperior part of the liver.

From this ſtate of the caſe, and from the appearances upon diſſection, I have little reaſon to believe that medicine could have procured any permanent relief. The ſudden death evidently dently proceeded from the rupture of the heart happening in that ventricle which anatomiſts have mentioned as moſt capable of making reſiſtance.

To what cauſe are we to attribute the preternatural attachment of the heart to the pericardium, and the depoſit of bone upon its ſurface? It may be urged that theſe changes muſt have been the reſult of previous inflammation. I can make but one objection to this hypotheſis, and that is, the abſence of pain. We well know that ſlight colds will often produce adheſion of the lungs to the pleura; but the change here took place in a part of much more exquiſite ſenſibility. The heart ſeemed to have experienced a morbid alteration, ſimilar to that which had pervaded the reſt of the muſcular ſyſtem. The ſymptoms above recited did not indicate the dropſy of the pericardium; and the ſlowneſs of the pulſe, as well as the abſence of rigors and colliquative evacuations, were circumſtances I have never before obſerved or read of in caſes of pulmonary ulceration.

Contrary to what we uſually find, this gentleman's reſpiration was invariably better in cold weather than when the atmoſphere was moderately warm.

Medical writers, in defcribing the fymptoms of hydropic effufion in the pericardium, lay it down as an eftablifhed maxim, that, in fuch cafes, there is an intermitting pulfe, and an inability to lay on the right fide; but in this patient no fuch fymptom occurred; and a palpitation, fimilar to that with which he was troubled, is obfervable in every phthifical patient after accelerated motion.

Comparing the progrefs of the difeafe, the fymptoms that attended it, and its iffue, to thofe pulmonary complaints we fo often meet with or read of, it will appear very remarkable; and though we may perhaps affign fome plaufible conjectures for a few particular changes the parts underwent, yet there are others which I believe the moft fpeculative theorifts will not pretend to decide on.

The action of violent coughing muft at all times produce a temporary change in the circulation; but this will be generally proportioned to the refiftance the veffels are capable of making. This admitted, we muft not wonder that Mr. Jones died fuddenly; for, independent of this caufe, and the weakened ftate of the heart, that vifcus muft have experienced additional tional diftrefs and embarraffment from the fluid with which I found it encumbered.

*Walfall,*

Nov. 6, 1786.

